# Genotoxicity Revaluation of Three Commercial Nitroheterocyclic Drugs: Nifurtimox, Benznidazole, and Metronidazole

**DOI:** 10.1155/2009/463575

**Published:** 2009-10-21

**Authors:** Annamaria Buschini, Lisa Ferrarini, Susanna Franzoni, Serena Galati, Mirca Lazzaretti, Francesca Mussi, Cristina Northfleet de Albuquerque, Tânia Maria Araújo Domingues Zucchi, Paola Poli

**Affiliations:** ^1^Dipartimento di Genetica, Biologia dei Microrganismi, Antropologia, Evoluzione, Università di Parma, Parco Area delle Scienze, 11/a, 43100 Parma, Italy; ^2^Departamento de Tecnologia Bioquímico Farmacêutica, Faculdade de Ciências Farmacêuticas, USP, Avenida Prof. Lineu Prestes, 580, Cidade Universitária, 05508-900 São Paulo, Brazil; ^3^Departamento de Parasitologia, Instituto de Ciências Biomédicas, USP, Avenida Prof. Lineu Prestes, 1374, Cidade Universitária, 05508-900 São Paulo, Brazil

## Abstract

Nitroheterocyclic compounds are widely used as therapeutic agents against a variety of protozoan and bacterial infections. However, the literature on these compounds, suspected of being carcinogens, is widely controversial. In this study, cytotoxic and genotoxic potential of three drugs, Nifurtimox (NFX), Benznidazole (BNZ), and Metronidazole (MTZ) was re-evaluated by different assays. Only NFX reduces survival rate in actively proliferating cells. The compounds are more active for base-pair substitution than frameshift induction in Salmonella; NFX and BNZ are more mutagenic than MTZ; they are widely dependent from nitroreduction whereas microsomal fraction S9 weakly affects the mutagenic potential. Comet assay detects BNZ- and NFX-induced DNA damage at doses in the range of therapeutically treated patient plasma concentration; BNZ seems to mainly act through ROS generation whereas a dose-dependent mechanism of DNA damaging is suggested for NFX. The lack of effects on mammalian cells for MTZ is confirmed also in MN assay whereas MN induction is observed for NFX and BNZ. The effects of MTZ, that shows comparatively low reduction potential, seem to be strictly dependent on anaerobic/hypoxic conditions. Both NFX and BNZ may not only lead to cellular damage of the infective agent but also interact with the DNA of mammalian cells.

## 1. Introduction

The introduction of nitro-substituted heterocyclic drugs, such as nitroimidazoles and nitrofurans, heralded a new era. 5-Nitrofurans and 2- and 5-nitroimidazoles are the classes of nitroheterocyclic drugs most used in the treatment of infections caused by anaerobic bacteria and a range of pathogenic protozoan parasites [1–4]. 

Nitrofuran derivatives do not interact with DNA per se, but they require metabolic conversion for exerting their action [5, 6]. These compounds are metabolically activated to the corresponding hydroxylamines through the reduction of their functional nitro group [7]. 

Nifurtimox (NFX, *N*-(3-methyl-1,1-dioxo-1,4-thiazinan-4-yl)-1-(5-nitro-2-furyl) methane-mine, CAS number 23256-30-6) is a 5-nitrofuran for the treatment of Chagas' disease (American trypanosomiasis), which affects more than 10 million people in Central and South America. Controversial results are reported for NFX in the treatment in the second stage of African sleeping sickness [8–10]. One possible mechanism of action of NFX as trypanocidal involves the ability of this agent to form a nitro-anion radical metabolite, which reacts with the nucleic acids of the parasite, causing a significant breakage in the DNA [4], and the other involves the production of superoxide anions and hence hydrogen peroxide [4, 11]. The drug exhibits particularly serious toxic side effects on patients [12, 13] generally attributed to NFX nitroreductive biotransformation to a nitroanion radical, redox cycling, generation of reactive oxygen species or lipid peroxidation [14, 15], production of peroxinitrite radicals [16], and other nitrite-forming processes [17]. Induction of mutants by NFX [18–24], but not by its in vivo metabolites [25], was found in bacterial systems. It exhibited genotoxic effects on *Drosophila melanogaster* [26], and it caused chromosomal aberrations (CA), micronucleus (MN) ,and sister-chromatid exchange (SCE) formation [27–29]. NFX was found to increase cancer incidence [30, 31] but did not exhibit initiating carcinogenic activities [32]. 

Nitroimidazol derivatives are reported to exert their therapeutic effect through nitrogroup reduction [2, 3]. Antibacterial and antiprotozoal activity appears to result from the formation of short-lived protonated one-electron nitro radical anions and other compounds, including nitroso and hydroxylamine derivatives [3] following reduction of the nitrogroup by nitroreductases present in the bacterial or protozoal cell. This intermediate species interacts with DNA, causing strand breaks with helix destabilization, this preventing DNA synthesis and, thereby, causing cell death in both bacteria and protozoal parasites [33, 34].

The 2-nitroimidazol benznidazole (BNZ, *N*-benzyl-2-(2-nitroimidazol-1-yl)acetamide, CAS number 22994-85-0) is the only etiological treatment commercially available for Chagas disease in many countries from Latin America. This drug appears to inhibit protein and ribonucleic acid (RNA) synthesis in the parasite. As NFX, BNZ induces serious toxic side effects [12]. Several free radical species similar to those produced by NFX were thought to be involved [4, 14]. It is mutagenic for *Salmonella typhimurium* [19, 20, 22, 33–36] as well as the urine and blood of treated animals [25]. Its clastogenic ability is controversial since both positive [27, 28, 37–43] and negative [44] findings are reported. Carcinogenicity bioassays reported cancer induction [30, 31]. 

The 2-nitroimidazol metronidazole (MTZ, 2-(2-methyl-5-nitro-1*H*-imidazol-1-yl)ethanol, CAS number 443-48-1) possesses direct trichomonacidal and amebacidal activity and shows clinical activity against most obligate anaerobes [1, 45]. The drug is active when reduced under strongly reducing conditions [46] through electron donation from ferredoxin or flavodoxin, oxidoreductase [47], and possibly forming an hydroxylamine [48]. Presumably, free radicals are formed which, in turn, react with cellular components resulting in death of the microorganism as also supported by some findings on the protective role of antioxidants against MTZ [49]. However, scavenging activities of MTZ on free oxygen radicals were reported [50–52]. It has been also suggested that the reactive species are intermediates in the reductive metabolism of MTZ, which leads to the formation of acetamide [53]. Mutagenic activity, induced by the drug [22] as well as by the urine of therapeutically treated patients [54, 55], was found in bacterial systems. In mammalian cells, some studies [33, 34, 56–65] indicate that MTZ causes a loss of DNA helix content, strand breakage, unscheduled DNA synthesis, and SCEs, whereas others [66–72] do not confirm its genotoxicity. In some in vivo studies, MTZ was found genotoxic [73, 74]. In many others [68, 71, 75–79] these effects were not registered. Some data [80] cannot be clearly interpreted. Some positive effects [57, 66] have been discussed [81] as to be the result of an infection of the cells with mycoplasms. According to the International Agency for Research on Cancer [82], the evidence is sufficient to consider MTZ as an animal carcinogen [83–87] but insufficiently for humans [88].

For chemotherapy of Chagas' disease, NFX and BNZ display most efficacy against the extracellular forms of *T*.* cruzi* during the acute phase of the infection, whereas both drugs are considered to be, at best, only partially beneficial against the intracellular form that causes chronic disease [89–94]. Furthermore, the severe side effects of both drugs limit their use [12, 13, 92–94]. MTZ is currently the drug of choice for treating invasive amoebiasis, but it may not be sufficient to eliminate parasite cysts in the intestine. Moreover, some unpleasant adverse effects associated with metronidazole in some patients, and the possibility of parasite resistance to metronidazole has to be considered [95]. Treatment of patients with *Clostridium difficile *infection with MTZ generally reduces morbidity and mortality, although the number of patients that do not respond is increasing [96–98]. There are insufficient data to recommend the use of metronidazole in persistent diarrhoea of unknown cause or nonspecific cause [99]. Furthermore, these compounds were suspected of being mutagens and carcinogens [3, 4, 7, 15, 27–29, 31, 33, 34, 94, 100].

The literature on these widely used drugs is controversial. To come to a more clear statement with respect to the mode of action we re-evaluated their genotoxic and cytotoxic potential with different in vitro test, that is, Salmonella reverse mutation assay [101], comet assay [102], micronucleus assay [103, 104], and short- and long-term cytotoxicity assays.

## 2. Materials and Methods

### 2.1. Chemicals

Reagents for electrophoresis, normal melting point and low melting point agarose, dimethyl sulfoxide (DMSO), ethidium bromide (EtBr), 5-carboxyfluorescein diacetate (FDA), Hoechst 33342 (HO), ethyl methane sulfonate (EMS), hycantone (HYC), 2-aminofluorene (2-AF), cell culture medium, buffers, and general laboratory chemicals were from Sigma (Sigma-Aldrich Company Ltd., Milan, Italy). Lyophilized postmitochondrial supernatant rat liver fraction S9 was from MOLTOX INC. (Boone, NC, USA); Bleomycin from Rhône-Poulenc Rorer (Collegeville, PE, USA); Furylfuramide (2-(2-furyl)-3-(5-nitro-2-furyl)acrylamide) from Wako Pure Chemical Industries (Osaka, Japan). Nifurtimox (Bayer 2502, Lampit) was from Bayer. Benznidazole (Rochagan) was from Roche Brasil (Rio de Janeiro, Brazil). Metronidazole was from Fluka Sigma-Aldrich (Buchs, Swiss) and Bieffe Medital SPA (Grosotto, SO, Italy).

### 2.2. Salmonella/Microsome Test

The bacteria reverse mutation assay [101] on different his^−^ *Salmonella typhimurium* strains was used to evaluate the mutagenic properties of the compounds. TA100 strain is predominantly sensitive to base pair substitution mutagens whereas TA98 is sensitive to frameshift mutagens. The strains are deficient in excision repair (uvrB mutation) and contain the plasmid pKM101, which activates an error prone DNA repair system, making them more responsive to a variety of mutagens. The test was performed in the absence or in the presence of exogenous metabolic activation system (Aroclor 1254-induced rat liver S9, prepared from adult Sprague Dawley rats, is supplemented with different cofactors (glucose-6-phosphate, NADP-Na^2^) to a final protein concentration of 2 mg/mL incubation) to detect indirect and direct mutagenic activity.

Compounds having a nitro function attached to an aromatic or heteroaromatic moiety constitute a group of chemicals biologically active. The reactive forms are metabolically generated through nitroreduction and, in many cases, through oxidative pathways: whereas the oxidative pathways depend on the presence of the cytochrome P450 family of enzymes and occur, therefore, mainly in the liver, nitroreduction is mainly found in bacterial cells [91]. This may explain why nitro compounds are generally strong mutagens in the *Salmonella *mutagenicity assay, whereas mutagenicity in mammalian cells is not always found for *Salmonella*-positive nitro compounds [105]. In this context, the standard plate incorporation procedure for detecting his^+^ revertants was integrated by nitroreductase deficient strains to better understand compound mechanisms. These bacteria, TA98NR and TA100NR strains, are lacking the “classical” nitroreductase and were isolated as niridazole resistant derivatives of TA98 and TA100 strains, respectively [106, 107].

The results of the *Salmonella* assays are given as mean number of revertants from three independent plates (±SD). The data were analysed by using SPSS 11 (SPSS Inc., Chicago, IL, USA) statistical package. A one-way analysis of the variance test was performed. If a significant *F* value (*P* ≤ .05) was obtained, the comparison between controls and treated samples was analysed by using Dunnett's *C*-test. A positive result was defined as a reproducible dose-related increase in the number of his^+^ revertants. Least squares linear regression analysis was used to calculate specific activity. The activity of the S9 mix and the responsiveness of the tester strains were verified by including appropriate controls into each experiment. Specifically, DMSO (80 *μ*L/plate), used to redissolve the compounds, was identified as the negative control whereas hycantone (HYC, 75 *μ*g/plate) and 2-aminofluorene (2-AF, 2.5 *μ*g/plate) were used as positive controls without and with S9 mix, respectively [108]. In order to characterize the mutagenic response of NR strains with respect to the parental strains, we examined their sensitivity to 2-nitrofluorene (2-NF, 15 *μ*g/plate) and furylfuramide (FYFA, 20 ng/plate), whose mutagenicity is substantially lower in nitroreductase deficient than in nitroreductase proficient strains [109].

### 2.3. Cytotoxicity Assays in Human Cells

**Short-Term Exposure**

Heparin-anticoagulated peripheral blood was obtained by venipuncture from consenting healthy nonsmoker donors as provided by the AVIS (Italian Association of Voluntary Blood Donors). In order to isolate the leukocytes, the blood was maintained at 37°C for 5 minuets in an erylysis buffer (155 mM NH_4_Cl, 5 mM KHCO_3_, 0.005 mM Na_2_EDTA, pH 7.4), centrifuged and washed with PBS, and finally resuspended (~10^6^ cells/mL) in RPMI-1640 medium (Gibco). Appropriate amounts of the compounds were added to an Eppendorf tube containing the cell suspension (10^6^ cells). The cells were treated for 1 hour at 37° and then washed twice in PBS. Toxicity was checked immediately after the exposure. Cell survival was determined by the carboxyfluorescein diacetate/ethidium bromide-assay added with Hoechst 33342 (HO). A freshly staining solution (15 *μ*g/mL carboxyfluorescein diacetate, 2.5 *μ*g/mL ethidium bromide, 2 *μ*g/mL HO in PBS) was prepared. 500 *μ*l of cell suspension (equivalent to about 5 × 10^5^ cells) was mixed with 10 *μ*L of the staining solution, maintained at 37°C for 5 minuets. The cells were counted (200 cells per data point) under a fluorescent microscope (DAPI/FITC filters): viable leukocytes, whose nucleus is blue-stained by Hoechst 33342, develop a cytoplasmic green fluorescence, while dead cells accumulate ethidium bromide to develop orange fluorescent DNA.

**Long-Term Exposure**

Immortalized lymphocytes, that is, peripheral blood cells transformed by Epstein-Barr virus in a lymphoblastoid cell line able to actively proliferate (kindly provided by Dr. Dolcetti, (Centro di Riferimento Oncologico, CRO Aviano, Italy), were used for this assay. The cells (2 × 10^5^ cell/mL were incubated at 37°C in an atmosphere containing 5% CO_2_ during 48 hours in a 96-well plate in the presence of different concentrations of the drug. At the end of the exposure period, cellular suspension was added with the solution reagent (20 *μ*L) of CellTiter 96 Aq_ueous_ One Solution Cell Proliferation Assay (Promega, Madison, WI, USA). After 4 hours incubation, the absorbance at 490 nm was recorded with a 96-well plate reader (MULTISKAN EX, Thermo Electron Corporation, Vantaa, Finland). The quantity of formazan product as measured by the absorbance at 490 nm was directly proportional to the number of living cells in culture [110, 111].

### 2.4. Comet Assay

#### 2.4.1. Alkaline Assay

The Comet assay was performed, basically according to Singh and coll [102] with minor modifications, on fresh human leukocytes treated as previously described for short-term cytoxicity assay (1hour, 37°C) [112]. Cell lysis was carried out at 4°C overnight by exposing cells to a buffer containing 2.5 M NaCl, 10 mM Na_2_EDTA, 10 mM Tris-HCl, 1% Triton X-100, and 10% DMSO, pH 10. DNA unwinding was achieved over 20 minutes in an electrophoretic alkaline buffer (1 mM Na_2_EDTA, 300 mM NaOH, 0°C, pH > 13); electrophoresis was then carried out for 20 minutes (0.78 V/cm, 300 mA) at 0°C in the same buffer, followed by neutralisation in 0.4 M Tris-HCl, pH 7.5. DNA was stained with 100 *μ*L ethidium bromide (2 *μ*g/mL) before the examination at 400x magnification under a Leika DMLB fluorescence microscope (excitation filter BP 515–560 nm, barrier filter LP 580 nm) using an automatic image analysis system (Release 2.1–Sarin, Florence, Italy). The migration distance between the edge of the comet head and end of the tail (total length, TL) provided representative data on genotoxic effects. The samples were coded and evaluated blind (50 cells per each of two replicate slides per data point). All of the tests were performed at least three times. Ethyl methane sulfonate (2 mM) was used as positive control (TL = 61.03 ± 4.73 *μ*m).

#### 2.4.2. Modified Comet Assay for Detection of Oxidised Bases

By using endonuclease III (ENDOIII), a DNA glycosylase/endonuclease able to recognise and cleave classes of lesions, specific DNA base modifications such as oxidised pyrimidine bases are converted to strand breaks. These strand breaks can be detected by the comet assay as previously reported. Oxidatively generated damage can be evaluated easily by comparing the DNA migration in enzyme-or buffer-treated samples. The comet assay, with the modification of an extra step after lysis in which DNA is digested with the repair enzyme, was performed according to Collins et al. [113]. Briefly, after cell lysis, the slides were washed three times with the enzyme buffer (0.1 M KCl, 0.5 mM Na_2_EDTA, 40 mM HEPES (4-(2-hydroxyethyl)-1-piperazineethanesulfonic acid), 0.2 mg/mL bovine serum albumin, pH 8 with KOH) and incubated with ENDOIII in this buffer (or in buffer alone). Hydrogen peroxide (50 *μ*M) was used as a positive control (TL = 47.13 ± 2.09 *μ*m). ENDOIII was isolated from bacteria containing over-producing plasmids (Collin's Laboratory, Rowett Research Institute, Bucksburn, Aberdeen, UK). The enzyme-treated gels reveal alkali labile sites and strand breaks (ALS/SB) and oxidised bases (ALS/SB + OX). Assuming a linear dose response, subtraction of (ALS/SB) from (ALS/SB + OX) gives a measure of oxidised bases.

The SPSS 11 statistical package was used to analyse statistical differences between samples. Statistical differences between controls and treated samples were first determined with the nonparametric Wilcoxon rank-sum test for each experiment. The mean values from the repeated experiments were used in a one-way analysis of variance. Analysis of variance was followed by single or multiple pairwise comparisons. Data were tested for normality and homogeneity of variance. When these criteria were met, the data were compared using Dunnett's version of the *t*-test.

### 2.5. Micronucleus (MN) Assay

The MN assay was performed using blood samples from healthy, nonsmoking males, as provided by the AVIS (Italian Association of Voluntary Blood Donors). Lymphocytes were separated by Lymphoprep density gradient (Axis-Shield PoC As, Oslo, Norway) and, after two washes in RPMI 1640 medium, cultured at a concentration of 5 × 10^5^ cells/mL in RPMI 1640 containing 15% fetal calf serum (FCS), 2% v/v KaryoMAX phytohemoagglutinin (Invitrogen LTD, Collegeville, PE, USA), 2 mM L-glutamine, 25 IU/mL penicillin, and 25 *μ*g/mL streptomycin. The cultures were incubated at 37°C in an atmosphere containing 5% CO_2_ for 72 hours. Cytochalasin-B was added 44 hours after the start of incubation at a final concentration of 6 *μ*g/mL; at 48 hours, the lymphocytes were treated with the different drugs. Sterile DMSO (1.6 *μ*l/ml) was used as a solvent control, and the positive controls were bleomycin (6 *μ*g/ml), ethyl methane sulfonate (120 *μ*g/ml), and demecolcine (0.5 *μ*g/ml). Each treatment was tested with cells from two donors, performed in duplicate separate cultures (i.e., four cultures were set up for each treatment). After incubation, the lymphocytes were collected and resuspended in a mild hypotonic solution (0.075 M KCl) and then added with an ice-cold 5 : 3 acetic acid : methanol solution. After centrifugation (500 g, 10 min), the pellets were resuspended in cold (−20°C) methanol and maintained at −20°C (at least 24 hours). The cells were 2 fold washed (7 : 1 methanol:acetic acid, −20°C), plated on cold degreased slides, air-dried, and then stained with 2% v/v Giemsa (Carlo Erba, Milano, Italy). Scoring was done using an Exacta-Optech (Munich, Germany) light microscope at 1000X magnification. Micronuclei were scored according to the criteria described by Fenech [103, 104]. At each dose, at least 1000 binucleated (BN) lymphocytes for each culture were examined for the presence of one or more micronuclei. The MN frequency in 1000 BN cells was then calculated for each treatment. Assays with too few BN cells to determine the MN frequency (due to extensive cytotoxic effects) were classified as “toxic.” Cell-cycle parameters were evaluated by classifying 1000 cells according to the number of nuclei. 1000 cells were counted and scored as mononucleated, binucleated, trinucleated, or tetranucleated and the percentages of the different types of cells were calculated. The nuclear division index (NDI) was calculated by the formula, NDI = (M1 + 2M2 + 3M3 + 4M4)/N, where M1 through M4 indicate the number of cells with 1/4 nuclei, and N indicates the total number of cells scored [114].

The statistical analysis of MN frequency was performed using the ^2^ *χ*-test. NDI data were analyzed by Student's *t*-test.

## 3. Results

### 3.1. Salmonella Plate Incorporation Test

The compounds were investigated on different strains (TA100, TA100NR, TA98, TA98NR) with/without microsomal rat liver fraction (S9 mix). The compounds are more active for base-pair substitution (TA100) than frame-shift induction (TA98). Mutant induction is poorly affected by S9 metabolic activation whereas the nitroreduction process is an essential requirement in order to induce mutagenic effects on Salmonella. On TA100 strain, NFX and BNZ seem to have a similar specific activity (Table 1). MTZ significantly increases the revertant number only at concentrations higher than NFX- and BNZ-effective ones (see Table 1 in Supplementary Material avalible online at doi : 10.1155/2009/463575) and its specific activity is more than ten times lower than NFX and BNZ (Table 1). NFX is the most active on TA98 strain, which shows a low sensitivity against BNZ and MTZ.

### 3.2. Human Cells

#### 3.2.1. Cytotoxicity Assays

**Short-Term Exposure**

For all the compounds, the cellular survival rate did not appear to be affected by the 1hours-treatment in the range of doses used (NFX: 0–348 *μ*M; BNZ: 0–348 *μ*M; MTZ:  0–585 *μ*M) when measured immediately after the exposure (data not reported).

**Long-Term Exposure**

**Figure 1:**
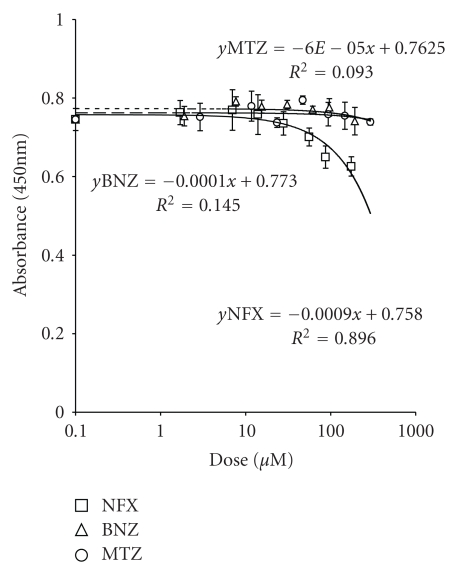
Cytotoxic effects induced in immortalized lymphocytes when detected by MTS assay (48 hours treatment).

The assay on cultured lymphocytes treated for 48 hours (Figure 1) did not show any significant effects induced by BNZ and MTZ whereas a significant decrease of cell population (*P* < .05) was observed for NFX at concentration ≥ 56 *μ*M. These cells need a 48-hour period for doubling their number. In this context, for evaluating if NFX cytotoxic effect was cell-cycle dependent, the cellular survival was also evaluated after a shorter exposure time (24 hours). In this case, no cytotoxic effect was induced (see Figure 1 in Supplementary Material).

#### 3.2.2. Genotoxicity Assays

**Comet Assay**

**Figure 2:**
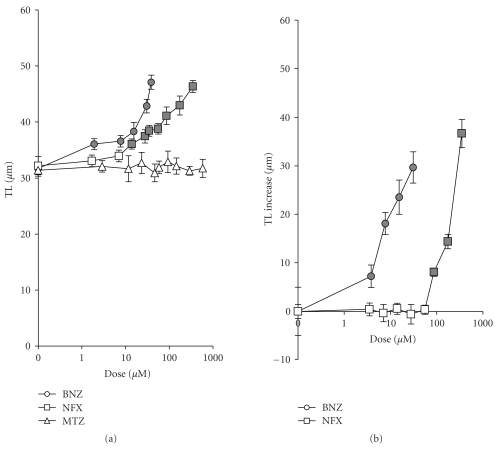
DNA damage detected in human leukocytes treated (37°C, 1 hour) with NFX, BNZ, and MTZ. (a): DNA damage is expressed as total migration length (TL, *μ*m) detected by the alkaline comet assay. (b) Specific oxidatively generated damage to DNA detected by the modified comet assay (ENDO III). DNA oxidative damage is expressed as DNA migration (*μ*m) increase with respect to the assay without enzymes. Mean ± SD of three independent experiments. Filled symbols, *P* < .05 (Dunnett's C) with respect to dose 0.

The data relative to the primary DNA damage detected on human leukocytes after 1-hours treatment by the comet assay performed at pH > 13 (Figure 2(a)) show strong differences among the three drug effectiveness. To improve our understanding on the drug action mechanism, we proceeded to specifically measure BNZ- and NFX-induced oxidative DNA damage by using bacterial repair endonuclease III in the modified comet assay protocol [113] (Figure 2(b)). BNZ DNA damaging activity was mainly linked to oxidative stress whereas NFX induced DNA base oxidation only at doses ≥ 87 *μ*M. These findings suggest that NFX could act by a dose-dependent mechanisms: at lower doses, a DNA damage basically independent from the oxidative stress, that is, DNA migration increase detected by the “classic” assay, and an oxidation of DNA bases at higher doses. BNZ specific activity (Table 2) in inducing DNA oxidative damage was about tenfold higher than that of NFX.

**MN Assay**

On 24-hours treated lymphocytes, the nitrofuran NFX was able to induce a significant increase (*P* < .05) of MN (Table 3) whereas the two nitroimidazoles (BNZ and MTZ) did not alter MN frequency in the range of doses used (see Table 2 in Supplementary Material). NFX was also the only drug that significantly (*P* < .05) reduced nuclear division (Table 3). The percentages of mono-, bi-, tri-, and tetranucleated cells in NFX-treated lymphocytes (see Figure 2 in Supplementary Material) well represents this reduction. Since on 24-hours treated lymphocytes, MTZ and BNZ did not induce any effect, the cells were exposed to the drugs for a longer time (72 hours) as previously reported for MTZ [115]. The data (Table 3) show an increase of MN frequency in cell treated with BNZ, without some decrease of nuclear division. On the other hand, the treatment with MTZ did not induce any alteration of both MN and NDI.

## 4. Discussion and Conclusions

The data obtained in this study on the *S. typhimurium* mutation assay show that the three drugs are more active for base-pair substitution than frameshift induction, in agreement with previous reports on nitroheterocycle compounds [7, 19, 20] and are widely dependent from nitroreductase activity. A study [19] reports that the presence of S9 mix decreased BNZ and NFX mutagenicity on Salmonella even if these data were not confirmed [20]. Our data show that microsomal fraction S9 does not or weakly alter the mutagenicity of the tested compounds. The three drugs are differently effective on Salmonella: NFX and BNZ induce in the TA100 strain a significant revertant increase at the lowest used dose whereas MTZ significantly increases the revertant frequency only at higher doses.

The data on Salmonella suggest that these drugs act as direct mutagens, as generally suggested for nitroheterocycle compounds [7]. On the other hand, nitrogroup reduction strongly influences their mutagenic capability. The DNA-reactive forms of nitro compounds may be metabolically generated through nitroreduction [4] and, in some cases, through oxidative pathways [105]. 

Bacterial mutagenicity assay could give an exaggerated picture of the potency of nitro compounds relative to the effects (mutation and tumour induction) observed in animals [7, 116, 117]. This is probably due to two factors: first, to the greater activity of the nitroreductases in bacteria compared to mammalian cells, and second, to the detoxification reactions and excretion processes which occur in animals. Indeed, these factors produce the difference in sensitivity between pathogen and host, which is the basis for the practical use of nitrocompound derivatives in clinical and veterinary medicine. This resulted in labelling the *Salmonella *mutagenicity assay as ‘not predictive' for this class of compounds [7, 116, 117]. The evaluation of the mutagenic potential of a nitrocompound, therefore, relies strongly on the outcome of tests conducted in mammalian systems.

In previous studies [62], MTZ produced significant dose-related increases of DNA damage, when tested on human lymphocytes in aerobic conditions using the comet assay. Concerning the mechanisms of action, these authors proposed that MTZ was able to induce DNA damage through the so-called futile cycle that is, one electron reduction of the drug leads to the production of nitroradical anions which are oxidised in the presence of oxygen and may generate reactive oxygen species (ROS). On the other hand, in our experiments, MTZ did not induce any significant increase on DNA migration up to 585 *μ*M, that is, about tenfolds higher (100 *μ*g/ml) than plasmatic concentration in MTZ-treated patients (8–13 *μ*g/ml). In actual fact, conflicting results are reported in literature regarding MTZ genotoxicity in mammal cells [33, 34, 57, 60–66, 68, 71–81]. The lack of any significant cytotoxic or genotoxic effects induced by MTZ in our experimental conditions on human cells suggests that MTZ biological activity could be strictly dependent on anaerobic conditions [81, 118]. In fact, Korbelik and Horvat [67] observed clastogenic effects only in hypoxic mammalian cells: in this condition, the toxic effect of a reduction of the nitro group should be similar to those in microorganisms living under anaerobic conditions. 

In agreement with some previous reports [27, 28, 37–39], our findings confirmed that BNZ and NFX are able to induce significant genotoxic effects on human cells. Furthermore, the lowest BNZ and NFX doses able to induce DNA damage detectable by the comet assay are in the range of plasmatic concentrations in patients therapeutically treated with these two nitro compounds. 

The two drugs are reported to produce similar free radicals [4, 14]. However, in our experimental conditions, the behaviour of BNZ and NFX in inducing DNA oxidative damage suggests different mechanisms of action for the two compounds. Specifically, the analysis of DNA oxidative damage data shows that BNZ mainly acts through free radical species production whereas dose-depending mechanisms could be suggested for NFX. Some differences between the nitrofuran and the nitroimidazol were also found in MN test: shorter exposure time is necessary for NFX than for BNZ (24 hours versus 72 hours) to induce a significant genetic damage.

Even cytotoxicity data suggest specific action mechanisms for the three compounds. Whereas no effect was observed on the survival of nondividing cells up to the highest doses used for all the drugs, NFX was able to induce cytotoxic effects on actively proliferating cells at 56 *μ*M. 

NFX and BMZ genotoxicity was detected by both comet and MN assay, even if in different ranges of concentrations. Comet assay showed significantly increased DNA damage at lower concentrations, but these lower concentrations were not able to increase the chromosomal damage detected by MN assay. The discrepancies between the results obtained by the Comet and MN tests, as reported in other investigations [119, 120], are probably caused by the nature of these two assays. The Comet detects primary DNA lesions resulting from the balance of DNA damage and repair mechanisms. The MN test detects DNA damage that has been transformed to structural and numerical chromosome alterations and reveals fixed DNA lesions or unrepairable aneugenic effects. 

A distinguishing property among the three compounds is their reduction potential. The 5-nitroimidazol derivative (MTZ) shows comparatively low reduction potential: it can be reduced to the corresponding amino derivative only under anaerobic conditions and its spectrum of activity can result limited to anaerobic or facultative anaerobic bacteria. On the other hand, both the antitrypanosomal drugs, NFX and BNZ, may not only lead to cellular damage of the infective agent but also interact with the DNA of mammalian cells by the formation of free radicals and redox cycling. 

In conclusion, the genotoxic profile of MTZ, NFX, and BNZ is further defined from our findings. MTZ therapeutic use could represent a genotoxic hazard only for the host cells in anoxic conditions. The high DNA damaging potential of NFX and BNZ on mammalian cells, together with other known severe side-effects on patients and low effectiveness in the second stage of the Chagas' disease, call for new safer drugs against *Trypanosoma* infection.

## Supplementary Material

Supplementary Table 1:
*Salmonella* plate incorporation test
Nifurtimox (NFX), Benznidazole (BNZ), and Metronidazole (MTZ) were investigated on different Salmonella strains (TA100, TA100NR, TA98, TA98NR) with/without S9 (Table 1). MTZ significantly increases the revertant number in TA100 only for concentrations ≥40 *µ*g/ml whereas the lowest effective dose of NFX and BNZ is 5 *µ*g/ml. NFX is the most active on TA98 strain, whereas BNZ and MTZ, that show a similar effectiveness, induce a significant revertant increase only at concentrations ≥ 250 *µ*g/plate.
Supplementary Figure 1:
Cytotoxicity assay
Long-term exposure - The assay on cultured lymphocytes treated for 48 hour by NFX shows a significant decrease of cell population (P<0.05) at concentration ≥16 *µ*g/m (Figure 1). These cells need a 48h period for doubling their number. In this context, for evaluating if NFX cytotoxic effect was cell-cycle dependent, the cellular survival was also evaluated after a shorter exposure time (24h). In this case, no cytotoxic effect was induced.
Supplementary Table 2:
Micronucleus (MN) Assay
On 24h-treated lymphocytes, the two nitroimidazoles (BNZ and MTZ) do not alter MN frequency in the range of doses used (Table 2). ), MTZ does not induce any alteration of both MN and ND also in cells treated for a longer time (72h).
Supplementary Figure 2:
NFX is the only drug that significantly reduced nuclear division: the percentage of mono, bi, tri, and tetranucleated cells in NFX-treated lymphocytes (Figure 2) well represents this reduction whereas no alteration with respect to the control is observed for BNZ and MTZ.

## Figures and Tables

**Table:** (a)

	Revertant increase/dose unit		
Strain	NFX	BNZ	MTZ
TA100	80.28 (0.99)	64.96 (0.98)	5.11 (0.98)
TA100 +	78.91 (0.98)	60.92 (0.98)	5.45 (0.96)
TA100NR	5.18 (0.99)	1.75 (0.81)	0.37 (0.94)
TA98	2.37 (0.99)	0.07 (0.88)	0.05 (0.89)
TA98 +	2.06 (0.99)	0.10 (0.93)	0.08 (0.97)
TA98NR	0.17 (0.94)	0.07 (0.93)	0.02 (0.79)

**Table:** (b)

	Revertant/plate			
Strain	HYC	2-AF	FYFA	2-NF
TA100	968 ± 103*	194 ± 21	1133 ± 70*	
TA100 +		521 ± 47*		
TA100NR	785 ± 97*		247 ± 34	
TA98	870 ± 90*	21 ± 5	189 ± 16*	169 ± 7*
TA98 +		808 ± 70*		
TA98NR	833 ± 71*		25 ± 4	19 ± 4

**Table 2:** Specific genotoxic activity of NFZ, BNZ, or MTZ on human leukocytes. I: alkaline comet assay; II: comet assay modified with ENDOIII for DNA oxidative damage.

	DNA migration/dose unit (*μ*m/*μ*M)	
Drug	I	II
NFX	0.0369 (*r* ^2^ = 0.8043)	0.106 (*r* ^2^ = 0.9732)
BNZ	0.3357 (*r* ^2^ = 0.941)	0.9077 (*r* ^2^ = 0.8351)
MTZ	0.0004 (*r* ^2^ = 0.017)	

**Table 3:** Mean frequency of Micronuclei (MN) in binucleated cells and Nuclear Division Index (NDI) in human lymphocytes treated with NFX (24 hours) or BNZ (72 hours) Positive controls:- ethyl methane sulfonate (1 *μ*M):15.0 ± 2.8; bleomycin (4 *μ*M): 26.0 ± 4.0; demecolcine (0.9 *μ*M): 65.0 ± 3.3.

***NFX (24 hours)***				***BNZ (72 hours)***			
**Dose**	NDI	NDI	MN	**Dose**	NDI	NDI	MN
**(** ***μ*** **M)**		(%)	(×10^−3^ BN)	**(** ***μ*** **M)**		(%)	(×10^−3^ BN)

**0**	1.97 ± 0.10	100	2.0 ± 0.5	**0**	1.93 ± 0.10	100	3.5 ± 0.5
**1.7**	1.95 ± 0.12	99 ± 6	2.5 ± 0.5	**1.9**	1.97 ± 0.10	102 ± 5	3.0 ± 0.5
**3.5**	1.97 ± 0.07	100 ± 4	2.0 ± 1.0	**3.8**	1.93 ± 0.11	100 ± 6	3.5 ± 1.0
**7.0**	1.89 ± 0.05	96 ± 3	3.0 ± 1.0	**7.7**	2.00 ± 0.07	104 ± 4	4.0 ± 1.0
**13.9**	1.85 ± 0.06	94 ± 3	2.5 ± 0.5	**15.4**	1.98 ± 0.06	102 ± 3	4.0 ± 1.5
**27.9**	1.75 ± 0.10	89 ± 5	3.0 ± 0.5	**30.7**	1.99 ± 0.05	103 ± 3	3.5 ± 1.0
**55.7**	1.69 ± 0.04^**a**^	86 ± 2^**a**^	3.0 ± 1.0	**61.5**	2.06 ± 0.07	107 ± 4	3.0 ± 0.5
**87.0**	1.61 ± 0.10^**a**^	82 ± 5^**a**^	4.0 ± 0.5	**96.1**	2.05 ± 0.03	106 ± 2	9.0 ± 0.5
**174.0**	1.43 ± 0.03^**a**^	73 ± 2^**a**^	9.0 ± 1.0	**192.1**	2.00 ± 0.07	104 ± 4	13.0 ± 1.0^**b**^
**348.1**	1.41 ± 0.02^**a**^	72 ± 1^**a**^	12.0 ± 1.0^**b**^	**384.2**	1.93 ± 0.10	100 ± 6	16.5 ± 0.5^**b**^

^**a**^ *P* < .05 Student's *t*-test; ^**b**^ *P* < .05^2^ *χ*-test
