# Supplementary material for: Genotoxicity Revaluation of Three Commercial Nitroheterocyclic Drugs: Nifurtimox, Benznidazole, and Metronidazole
Source: J Parasitol Res. 2009 Oct 21;2009:463575. doi: 10.1155/2009/463575 (PMC2963127; doi:10.1155/2009/463575)
Supplement: Supplementary file 1 — Supplementary Table 1: Salmonella plate incorporation test Nifurtimox (NFX), Benznidazole (BNZ), and Metronidazole (MTZ) were investigated on different Salmonella strains (TA100, TA100NR, TA98, TA98NR) with/without S9 (Table 1). MTZ significantly increases the revertant number in TA100 only for concentrations ≥40 µg/ml whereas the lowest effective dose of NFX and BNZ is 5 µg/ml. NFX is the most active on TA98 strain, whereas BNZ and MTZ, that show a similar effectiveness, induce a significant revertant increase only at concentrations ≥ 250 µg/plate. Supplementary Figure 1: Cytotoxicity assay Long-term exposure - The assay on cultured lymphocytes treated for 48 hour by NFX shows a significant decrease of cell population (P<0.05) at concentration ≥16 µg/m (Figure 1). These cells need a 48h period for doubling their number. In this context, for evaluating if NFX cytotoxic effect was cell-cycle dependent, the cellular survival was also evaluated after a shorter exposure time (24h). In this case, no cytotoxic effect was induced. Supplementary Table 2: Micronucleus (MN) Assay On 24h-treated lymphocytes, the two nitroimidazoles (BNZ and MTZ) do not alter MN frequency in the range of doses used (Table 2). ), MTZ does not induce any alteration of both MN and ND also in cells treated for a longer time (72h). Supplementary Figure 2: NFX is the only drug that significantly reduced nuclear division: the percentage of mono, bi, tri, and tetranucleated cells in NFX-treated lymphocytes (Figure 2) well represents this reduction whereas no alteration with respect to the control is observed for BNZ and MTZ. [file 463575.f1.pdf]

SUPPLEMENTARY DATA

Table I – Revertants induced by Nifurtimox (NFX), Benznidazole (BNZ), and Metronidazole (MTZ) in different *Salmonella* strains, with (+) or without (-) S9 mix. Mean (±SD) of three plates. Compound molarities were calculated taking into account the top-agar volume (2.5ml).

	Dose (µg/plate)	Dose (µM)	TA100 -S9	TA100 +S9	TA100NR -S9	TA98 -S9	TA98 +S9	TA98NR -S9
**NFX**	0	0	170±25	176±17	179±5	15±1	16±3	16±5
	5	7.0	618±10*	647±21*	224±8	30±4	32±4	16±2
	10	13.9	1100±24*	1145±13*	234±12	39±8	40±5	17±4
	20	27.8	1771±27*	1754±60*	302±10*	70±6*	52±4*	20±4
	40	55.7	2023±28*	1982±60*	366±11*	126±10*	105±4*	25±5
	80	111.4	1048±62*	1443±107*	608±33*	203±9*	181±8*	28±7
**BNZ**	0	0	171±10	180±15	183±7	17±4	21±2	22±2
	5	7.7	498±25*	406±13*	247±19	10±2	20±2	24±3
	10	15.4	943±27*	811±55*	249±6	16±4	18±2	28±2
	20	30.7	1801±67*	1445±85*	233±6	14±6	22±8	23±3
	40	61.5	3344±104*	3117±169*	318±9*	15±1	24±5	24±5
	80	123.0	5287±81*	4901±166*	343±26*	14±2	23±4	27±3
	125	192.1	nd	nd	nd	26±3	34±2	30±3
	250	384.2	nd	nd	nd	39±3	43±9	46±9
	500	768.5	nd	nd	nd	45±5	44±6	54±11
**MTZ**	0	0	196±20	180±18	205±3	16±3	21±5	21±7
	5	11.7	265±11	221±13	217±8	17±5	24±3	17±4
	10	23.4	258±24	273±24	190±23	20±3	19±5	20±4
	20	46.7	326±11	342±18*	216±12	18±4	21±5	19±1
	40	93.5	359±18*	418±20*	217±24	15±4	25±3	18±2
	80	187.0	551±56*	576±14*	236±12	18±2	30±6	17±2
	125	292.1	1053±19*	1185±37*	256±31	24±1	34±1	22±5
	250	584.3	1725±84*	1952±40*	331±46	32±1*	45±4	23±4
	500	1168.6	2666±168*	2777±91*	376±32*	39±2*	64±4*	27±5

* $p<0.05$ Dunnett's *C*
nd: not determined

Table II - Mean frequency of Micronuclei (MN) in binucleated cells (BN) and Nuclear Division Index (NDI) in human lymphocytes treated for 24 or 72 hours with BNZ, and MTZ

	Dose (μM)	NDI	NDI (%)	MN ($x10^{-3}$ BN)
BNZ (24h)	0	2.10±0.10	100	3.0±1.0
	1.9	1.99±0.10	95±5	3.0±0.5
	3.8	1.97±0.11	94±5	4.0±0.5
	7.7	1.97±0.07	94±3	3.5±1.0
	15.4	2.00±0.06	95±3	2.5±0.5
	30.7	2.04±0.05	97±2	4.5±1.0
	61.5	1.99±0.07	95±3	3.5±0.5
	96.1	2.01±0.03	96±1	3.0±0.5
	192.1	2.07±0.07	99±3	5.0±1.0
	384.2	2.04±0.10	97±5	3.5±0.5
MTZ (24h)	0	2.19±0.14	100	3.5±1.5
	2.9	2.04±0.12	104±6	2.5±0.5
	5.9	1.97±0.07	100±4	3.0±1.0
	11.7	1.97±0.05	100±3	2.0±0.5
	23.4	2.00±0.06	102±3	2.5±0.5
	46.8	2.01±0.10	102±5	3.0±0.5
	93.6	1.99±0.04	101±2	4.0±0.5
	146.2	1.97±0.10	100±5	3.0±1.0
	292.4	2.01±0.03	102±2	4.0±1.0
	584.8	1.94±0.06	98±3	3.0±1.0
MTZ (72h)	0	1.74±0.19	100	4.0±1.0
	2.9	1.74±0.11	100±6	3.5±0.5
	5.9	1.75±0.17	101±10	5.0±1.0
	11.7	1.74±0.10	100±6	4.5±1.0
	23.4	1.70±0.06	98±4	4.0±1.5
	46.8	1.81±0.09	104±5	4.5±0.5
	93.6	1.73±0.08	100±5	3.0±0.5
	146.2	1.84±0.11	106±6	4.0±1.0
	292.4	1.76±0.07	102±4	4.0±1.5
	584.8	1.74±0.12	100±7	5.0±1.0

Positive controls - ethyl methane sulfonate (120μg/ml): 15.0±2.8;
bleomycin (6μg/ml): 26.0±4.0;
demecolcine (0.3μg/ml): 65.0±3.3

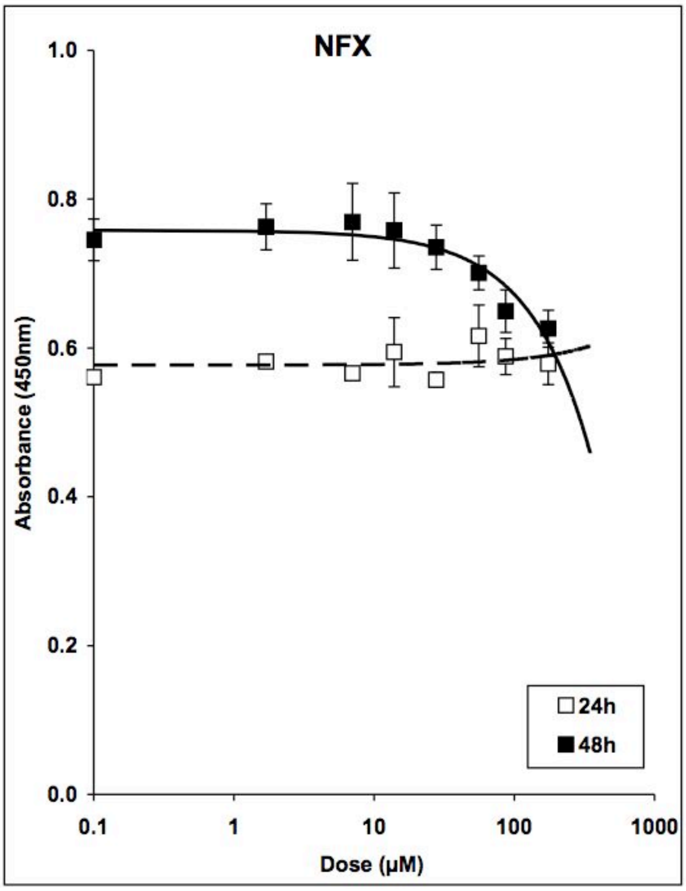

Figure I – Cytotoxic effects induced in immortalized NFX 24h- or 48h-treated lymphocytes when detected by MTS assay.

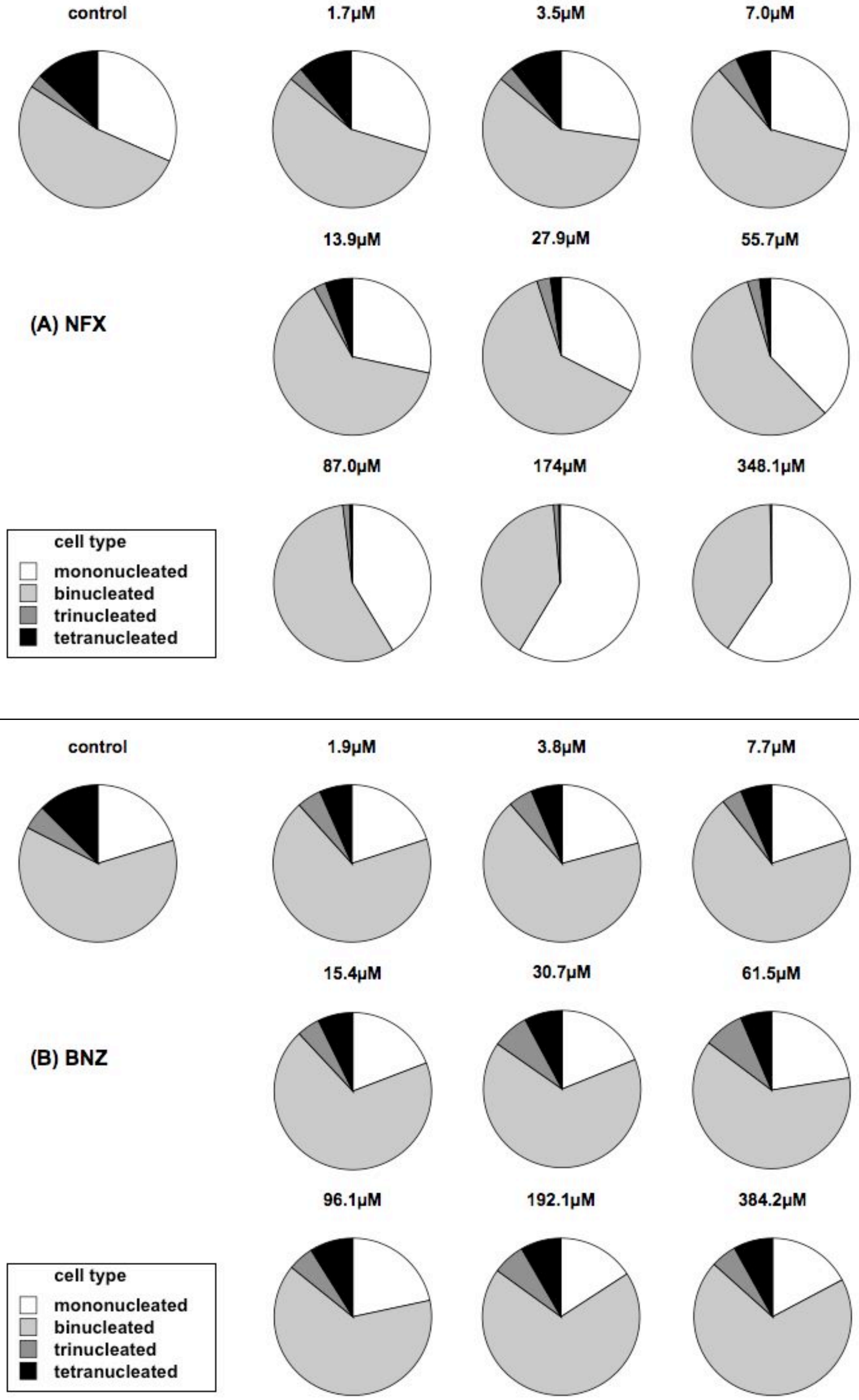
control
1.7μM
3.5μM
7.0μM
13.9μM
27.9μM
55.7μM
(A) NFX
87.0μM
174μM
348.1μM
cell type
mononucleated
binucleated
trinucleated
tetranucleated
control
1.9μM
3.8μM
7.7μM
15.4μM
30.7μM
61.5μM
(B) BNZ
96.1μM
192.1μM
384.2μM
cell type
mononucleated
binucleated
trinucleated
tetranucleated

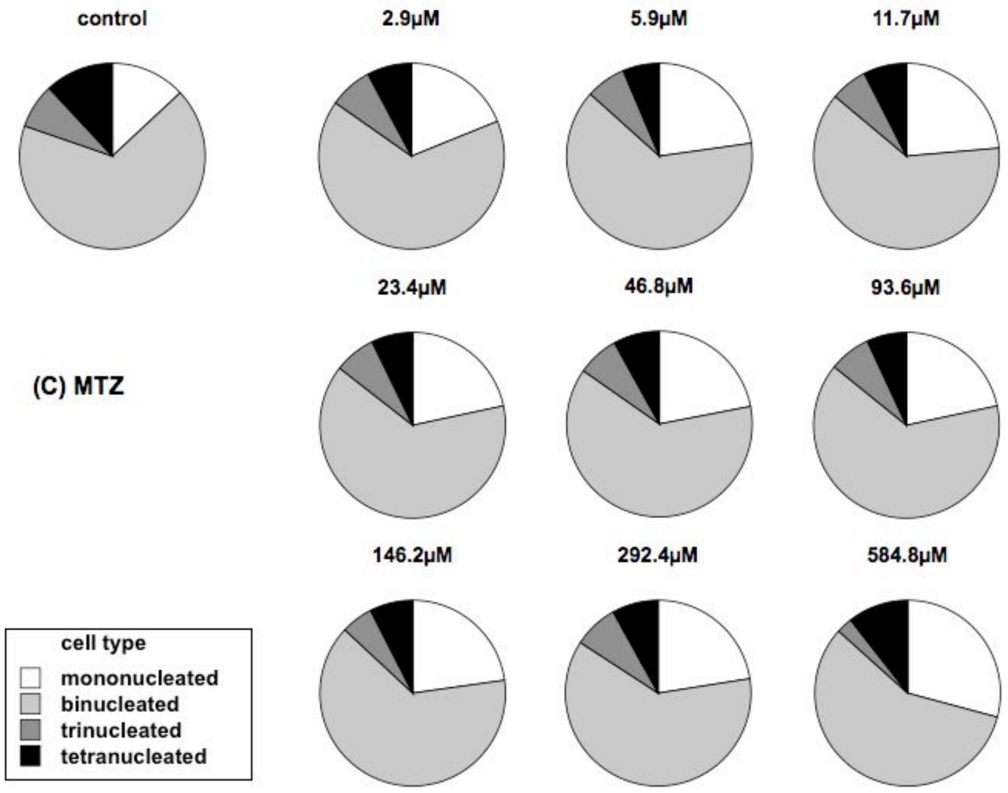

Figure II – MN assay. Percentage of mono-, bi-, tri-, tetranucleated cells within 24h-treated human lymphocytes with NFX (A), BNZ (B), and MTZ (C).
